# Selective Estrogen Receptor Modulators’ (SERMs) Influence on *TET3* Expression in Breast Cancer Cell Lines with Distinct Biological Subtypes

**DOI:** 10.3390/ijms25168561

**Published:** 2024-08-06

**Authors:** Kinga Linowiecka, Justyna Szpotan, Marlena Godlewska, Damian Gaweł, Ewelina Zarakowska, Daniel Gackowski, Anna A. Brożyna, Marek Foksiński

**Affiliations:** 1 Department of Human Biology, Institute of Biology, Faculty of Biological and Veterinary Sciences, Nicolaus Copernicus University in Toruń, Lwowska 1, 87-100 Toruń, Poland; j.szpotan@wp.pl (J.S.); anna.brozyna@umk.pl (A.A.B.); 2 Department of Clinical Biochemistry, Faculty of Pharmacy, Collegium Medicum in Bydgoszcz, Nicolaus Copernicus University in Toruń, Karłowicza 24, 85-092 Bydgoszcz, Poland; ewelinaz@cm.umk.pl (E.Z.); danielg@cm.umk.pl (D.G.); 3 Department of Cell Biology and Immunology, Centre of Postgraduate Medical Education, Marymoncka 99/103, 01-813 Warsaw, Poland; marlena.godlewska@cmkp.edu.pl (M.G.); damian.gawel@cmkp.edu.pl (D.G.)

**Keywords:** DNA demethylation, breast cancer, tamoxifen, 5-methylcytosine, 5-hydroxymethylcytosine, TET proteins

## Abstract

Tamoxifen, a selective estrogen receptor modulator (SERM), exhibits dual agonist or antagonist effects contingent upon its binding to either G-protein-coupled estrogen receptor (GPER) or estrogen nuclear receptor (ESR). Estrogen signaling plays a pivotal role in initiating epigenetic alterations and regulating estrogen-responsive genes in breast cancer. Employing three distinct breast cancer cell lines—MCF-7 (ESR+; GPER+), MDA-MB-231 (ESR−; GPER−), and SkBr3 (ESR−; GPER+)—this study subjected them to treatment with two tamoxifen derivatives: 4-hydroxytamoxifen (4-HT) and endoxifen (Endox). Through 2D high-performance liquid chromatography with tandem mass spectrometry detection (HPLC-MS/MS), varying levels of 5-methylcytosine (5-mC) were found, with MCF-7 displaying the highest levels. Furthermore, TET3 mRNA expression levels varied among the cell lines, with MCF-7 exhibiting the lowest expression. Notably, treatment with 4-HT induced significant changes in TET3 expression across all cell lines, with the most pronounced increase seen in MCF-7 and the least in MDA-MB-231. These findings underscore the influence of tamoxifen derivatives on DNA methylation patterns, particularly through modulating TET3 expression, which appears to be contingent on the presence of estrogen receptors. This study highlights the potential of targeting epigenetic modifications for personalized anti-cancer therapy, offering a novel avenue to improve treatment outcomes.

## 1. Background 

Epigenetic changes are crucial in the regulation of gene expression. One of the most common epigenetic mechanisms is linked with methyl group attachment to cytosine—DNA methylation [1]. Methyl groups are transferred by DNA methyltransferases (DNMTs) from S-adenosyl-L-methionine to the fifth carbon of cytosine [2]. DNA methylation marks are preserved during DNA replication via DNMT1 in the maintenance methylation process [3], yet they can still occur spontaneously via de novo methylation through the action of DNMT3a and DNMT3b [4]. If DNMTs are not present or unable to function, passive DNA demethylation occurs. However, in the last two decades, we have been conscious of an active DNA demethylation mechanism, which involves the ten-eleven-translocation (TET) family proteins. TET-dependent demethylation contributes to DNA hypomethylation through the oxidation of 5-methylcytosne (5-mC) to 5-hydroxymethylcytosine (5-hmC) [5]. 5-hmC can be subsequently further transformed to 5-formylcytosine (5-fC) and 5-carboxylcytosine (5-caC) [6]. The latter modified bases are excised in a base excision repair (BER) process via recognition of thymine DNA glycosylase (TDG) and replaced by unmodified cytosine [7,8]. Therefore, TDG is a crucial factor supporting the mechanism of active DNA demethylation. Although DNA methylation processes are essential phenomena during cell development and differentiation [9], they can be modulated by environmental factors following a random mutation in key genes involved in cell metabolism [10]. Evidence from the literature has emerged that epigenetic changes in the transcription of genes involved in the cell cycle, apoptosis, and cell growth are frequent in breast cancer [11,12,13,14,15]. Breast cancer is the most commonly diagnosed cancer in women worldwide, with more than 2 million new cases every year [16]. The majority of breast cancer cases are hormonal-dependent, which express estrogen and/or progesterone receptors [17]. The percentage of hormone-receptor-positive cells within a biopsy determine the treatment strategy for breast cancer patients [18]. Tamoxifen, a prominent drug in breast cancer hormonal therapy, is classified as a selective estrogen receptor modulator (SERM) [19]. It can function as either an estrogen agonist or antagonist, depending on whether it binds to the G-protein-coupled estrogen receptor (GPER) or the estrogen nuclear receptor (ESR) [20,21]. Estrogen can induce epigenetic changes in breast cancer [22], and estrogen-dependent genes are also subject to epigenetic regulation in breast cancer cells [23]. Additionally, epigenetic processes, such as DNA methylation, can influence the regulation of genes involved in drug responses and targets [24]. Therefore, tamoxifen’s pharmacological activity may extend beyond merely modulating estrogen signaling. This study aims to investigate whether tamoxifen, a representative of selective estrogen receptor modulators (SERMs), can induce changes in DNA methylation patterns in breast cancer cells with varying levels of GPER and ESR expression. As epigenetic markers are flexible and mostly associated with the response to hyper– or hypomethylating agents, the evaluation of 5-mC and its derivatives, as well as genes/proteins involved in the active DNA demethylation process, will provide insights as to whether the investigated modifications act as predictive markers as a result of SERMs treatment. 

## 2. Results

### 2.1. SERM’s Impact on the Proliferation Ratio of Breast Cancer Cells with or without Estrogen Receptors

**Figure 1:**
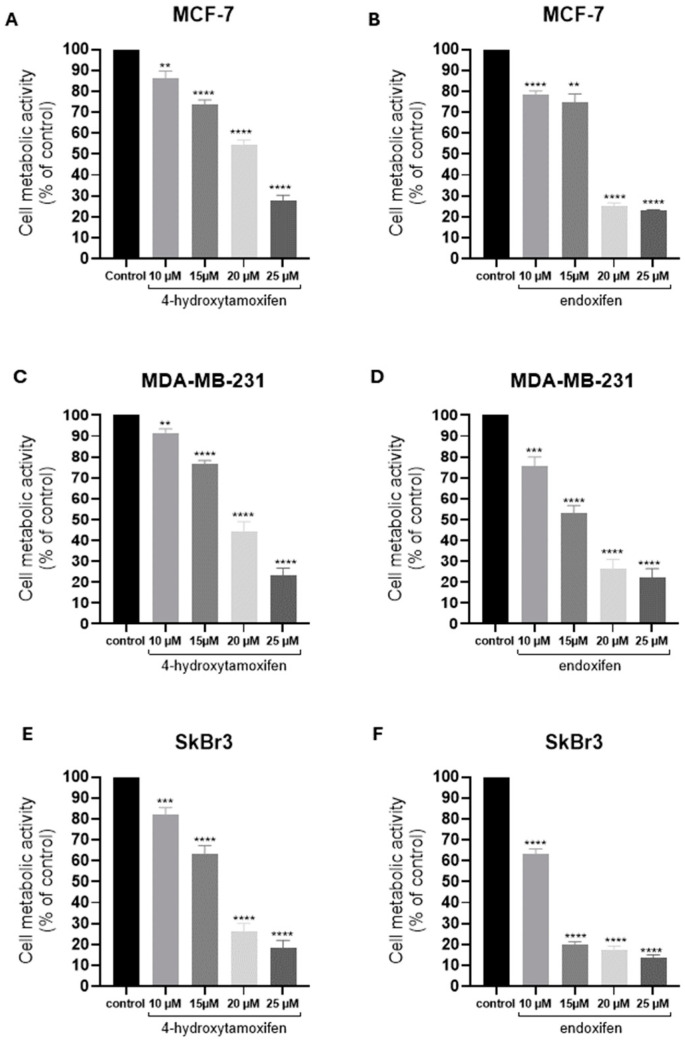
Cell metabolic activity after 4-hydroxytamoxifen (4-HT) and endoxifen (Endox) treatment in comparison to non-treated control cells. (**A**) MCF-7 cell metabolic activity after treatment with 4-HT; (**B**) MCF-7 cell metabolic activity after treatment with Endox; (**C**) MDA-MB-231 cell metabolic activity after treatment with 4-HT; (**D**) MCF-7 cell metabolic activity after treatment with Endox; (**E**) SkBr3 cell metabolic activity after treatment with 4-HT; (**F**) SkBr3 cell metabolic activity after treatment with Endox. ** *p* < 0.01; *** *p* < 0.001; **** *p* < 0.0001.

All breast cancer cell lines used in the study were treated with different concentrations of active tamoxifen derivatives: 4-hydroxytamoxifen (4-HT) and endoxifen (Endox) (Figure 1). The obtained results revealed a significant decrease in cell viability in all cell lines using an MTT-based assay. The reduction in cell viability after 4-HT ranged from 12 to 75% (MCF-7; Figure 1A), 7 to 81% (MDA-MB-231; Figure 1C), and 19 to 81% (SkBr3; Figure 1E). After Endox treatment, the reduction ranged from 20 to 78% (MCF-7; Figure 1B), 29 to 82% (MDA-MB-231; Figure 1D), and 38 to 86% (SkBr3; Figure 1F). Based on cell viability data, we chose 10 µM (for SkBr3) or 15 µM (for MCF-7 and MDA-MB-231) of 4-HT or Endox for subsequent experiments. 

### 2.2. TET3 mRNA Expression Was the Lowest in the Breast Cancer Cell Line with ESR and GPER Receptors

**Figure 2:**
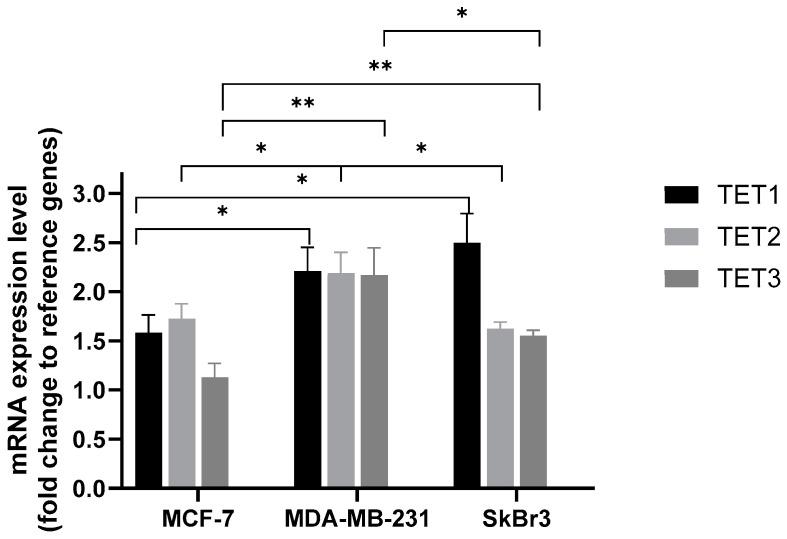
Comparison of *TET1*, *TET2*, and *TET3* mRNA expression level in MCF-7, MDA-MB-231, and SkBr3 cell lines. Significantly statistical differences: * *p* < 0.05: *TET1* MCF-7 vs. *TET1* SkBr3; *TET2* MCF-7 vs. *TET2* MDA-MB-231; *TET2* MDA-MB-231 vs. *TET2* SkBr3; *TET3* MDA-MB-231 vs. *TET3* SkBr3; ** *p* < 0.01: *TET3* MCF-7 vs. *TET3* MDA-MB-231; *TET3* MCF-7 vs. *TET3* SkBr3.

We observed significant differences in *TET1*, *TET2*, and *TET3* mRNA expression levels in all analyzed cell lines (Figure 2). *TET1* mRNA expression was the highest in the SkBr3 cell line, whereas the lowest was observed in the MCF-7 cell line. The highest expression level of *TET2* was observed in the MDA-MB-231 cell line. However, the most prominent result was the lowest level of *TET3* expression in the MCF-7 cell line. 

### 2.3. SERMs’ Impact on Expression of Genes Involved in Active DNA Demethylation in Breast Cancer Cells

**Figure 3:**
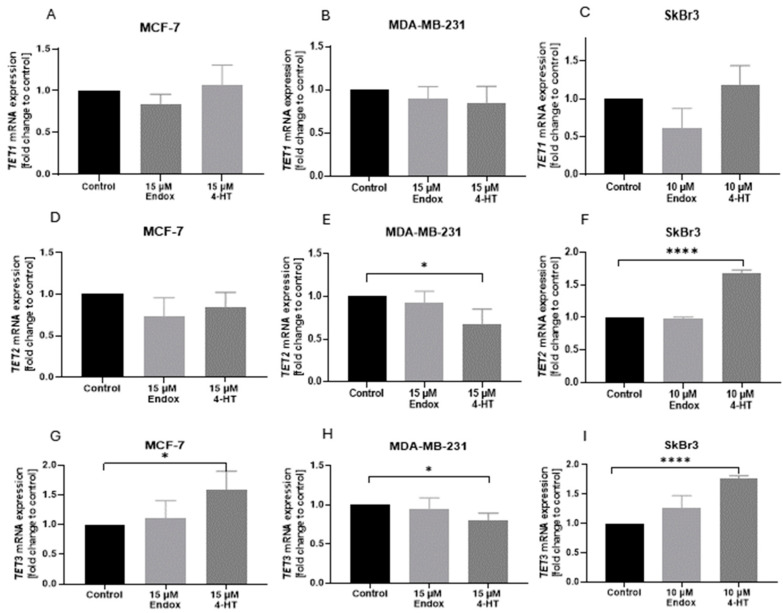
Comparison of (**A**–**C**) *TET1*, (**D**–**F**) *TET2*, and (**G**–**I**) *TET3* mRNA expression levels in MCF-7, MDA-MB-231, and SkBr3 cell lines after treatment with 4-HT and Endox. * *p* < 0.05; **** *p* < 0.0001.

Our study revealed, for the first time, the level of mRNA expression of *TETs* in breast cancer cell lines with differential expression of estrogen receptors after exposure to tamoxifen metabolites (Figure 3). We found that only one of the active derivatives of tamoxifen—4-HT—can significantly change the *TET* mRNA expression level. The *TET3* mRNA expression level was significantly increased after treatment with 4-HT in cells with expression of ER and GPER receptors (Figure 3G,I), contrary to the triple-negative breast cancer cells (Figure 3H). Additionally, a similar effect of 4-HT treatment was observed in *TET2* mRNA expression: it increased in SkBr3 cells (Figure 3F) and decreased in MDA-MB-231 (Figure 3E). The mRNA expression level of *TET1* did not significantly change after treatment with tamoxifen derivatives. 

### 2.4. The Highest Methylation Level Was Observed in Breast Cancer Cells with Estrogen and Progesterone Receptors

**Figure 4:**
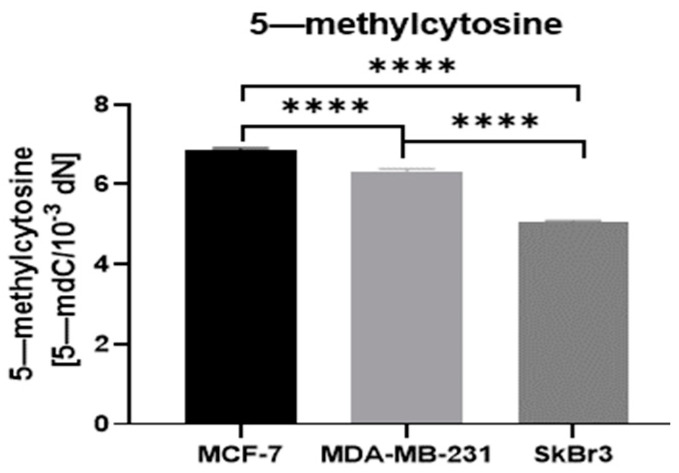
Comparison of 5-methylcytosine (5-mC) level in non-treated MCF-7, MDA-MB-231, and SkBr3 cell lines. **** *p* < 0.0001.

Analysis of 5-mC revealed a distinct methylation level in analyzed breast cancer cells. The highest level of 5-mC was detected in MCF-7 cells with expression of ER and progesterone (PGR) receptors. The triple-negative breast cancer cell line (MDA-MB-231) exhibited lower levels of 5-mC than MCF-7. The lowest 5-mC level was observed in the SkBr3 cell line with GPER receptors (Figure 4).

### 2.5. SERM Treatment Can Influence DNA Methylation Changes in Breast Cancer Cells’ Different Expression of Hormonal Receptors

**Figure 5:**
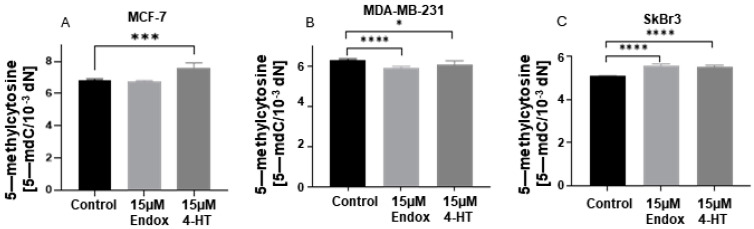
Comparison of 5-mC level in MCF-7 (**A**), MDA-MB-231 (**B**), and SkBr3 (**C**) cell lines after treatment with 4-HT and Endox. * *p* < 0.05; *** *p* < 0.001; **** *p* < 0.0001.

Treatment with 4-HT made a distinct impact on 5-mC level in different breast cancer cell lines. Breast cancer cells with estrogen, progesterone (MCF-7), or G-protein-coupled receptors (SkBr3) showed significantly higher levels of 5-mC after treatment with 4-HT (Figure 5A,C). Contrastingly, decreased 5-mC levels were observed in the triple-negative breast cancer cell line (MDA-MB-231) (Figure 5B). Treatment with Endox decreased the 5-mC level in MDA-MB-231 and increased it in SkBr3 (Figure 5B,C). Endox did not generate changes in MCF-7 (Figure 5A).

**Figure 6:**
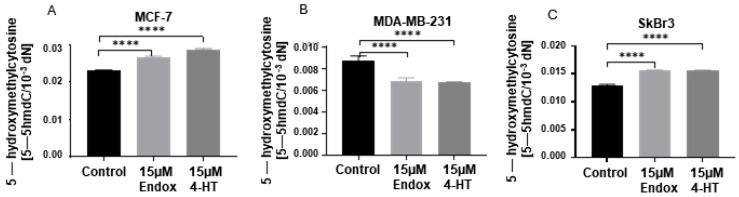
Comparison of 5-hydroxymethylcytosine (5-hmC) level in MCF-7 (**A**), MDA-MB-231 (**B**), and SkBr3 (**C**) cell lines after treatment with 4-HT and Endox. **** *p* < 0.0001.

Treatment with 4-HT also increased the level of 5-hmC in MCF-7 and SkBr3 cell lines (Figure 6A,C), whereas a decrease was observed in MDA-MB-231 cells (Figure 6B). Moreover, this 5-mC derivative also changed significantly in all analyzed cell lines after Endox treatment (Figure 6). 

## 3. Discussion

Epigenetic alterations are proven to be associated with carcinogenesis. Global DNA hypomethylation is considered a common marker of cancer; however, some promoter regions of genes can be hypermethylated [25]. Abnormal epigenetic changes are implicated in breast cancer tumorigenesis as well as progression and therapy response through various mechanisms, such as DNA repair, cell cycle aberrations, or hormonal regulation [26,27,28]. In addition, the characteristic methylation profiles at CpG dinucleotides were revealed as breast cancer risk factors associated with age, lifestyle, or reproductive characteristics [29,30,31,32,33]. Breast cancer is divided into five molecular subtypes according to different gene expression profiles [34]. The study by Holm et al. confirmed that different molecular subtypes harbor specific methylation profiles [35]. They also observed that typically 31% of CpG sites in breast tumors are hypermethylated, with a specific distinction between basal-like, Luminal A, and Luminal B subtypes [35]. In our study, we confirmed that different breast cancer cell lines, representing various subtypes of breast cancer, are associated with differential methylation patterns based on levels of 5-mC (Figure 4). Higher 5-mC levels were observed in breast cancer cell lines with expression of estrogen and progesterone receptors (MCF-7) in comparison to a triple-negative cell line (MDA-MB-231) or GPER-positive cell line (SkBr3). This is consistent with Holm et al., where they demonstrated a higher methylation frequency in Luminal B and Luminal A breast cancer subtypes [35]. Luminal B breast cancer was also indicated as a subtype with higher methylation density in a study conducted by Bediaga et al. [36]. Thus, 5-mC may be a useful marker to predict the breast cancer subtype, followed by its therapy response and outcome. Our study revealed a distinct *TET* mRNA expression level in analyzed cell lines. Interestingly, the expression levels of all *TETs* were higher in breast cancer cell lines without estrogen and progesterone receptors (MDA-MB-231 and SkBr3), which may explain the low level of 5-mC in these cell lines (Figure 2). TET proteins may use 5-mC for DNA demethylation processes; thus, the level of this derivative decreases. The highest level of *TETs* expression was found in MDA-MB-231 cells; however, the expression levels of all *TET* genes were similar in this cell line. The highest expression of *TET1* was found in the SkBr3 cell line, and the lowest expression of *TET3* was found in the MCF-7 cell line (Figure 2). Moreover, *TET2* expression was the highest amongst all *TET* genes in the MCF-7 cell line. These findings are consistent with a previous study by Sant et al., where they found an identical pattern of TET family expression levels in the MCF-7 cell line [37]. The lowest expression level of *TET3* in MCF-7 was also observed in a study by Yu et al. [38]. As detailed in our previous paper [39], *TET3* might have an ambiguous role in breast carcinogenesis. Previous studies have reported that *TET3* expression is decreased in breast cancer tissues and the MCF-7 cell line in comparison to heathy tissue counterparts and the HLB-100 breast cell line, respectively [40,41]. However, in contrast to those findings, expression of *TET3* is increased in mononuclear cells from peripheral blood obtained from breast cancer patients compared to healthy subjects’ cells [39,42]. It has been suggested that transcription regulators may bind directly to TET3, which regulates the 5-mc/5-hmC status in target gene promoters [43]. If such a regulatory mechanism exists, transcription-factor-dependent control of TET3 activity could potentially contribute to the epigenetic regulation of gene expression in cells. Other data have shown that inhibition of TET3 leads to the loss of 5-hmC in gene promoters [44]. 5-hmC via TET3 can regulate the transcription of genes related to the AMPK pathway, and thus TET3 can promote the proliferation, migration, and invasion of thyroid cancer [45]. Other studies indicate that TET3 can interact with nuclear receptors [46,47], thereby influencing the expression of many genes. Moreover, decreased TET3 level in the MCF-7 cell line may be associated with a potential role of estrogens in the modulation of *TET3* expression, as interaction between TET3 and estrogen receptor α (ESRα) has been reported [48]. 

ESRα, together with FOXA1 and GATA3, plays a crucial role in the regulation of luminal lineage specification and the direction of the endocrine response in breast tissue [49,50,51,52,53]. We found that in the ESR (+) and PGR (+) cell line (MCF-7), expression of *TET2* was higher in comparison to other *TET* genes. A mouse study revealed that *Tet2* haploinsufficiency leads to prominent changes in phenotype and a decreased 5-hmC level associated with ESRα repression [54]. TET2, in contrast to TET1 and TET3, is deficient in the CXXC DNA-binding domain and is instead recruited to particular chromatin regions by interacting with DNA-binding cell-specific transcription factors [55,56,57]. Thus, it was observed that TET2 can form a complex with FOXP1 that can mediate luminal cell differentiation through the coordination of ESRα, FOXA1, and GATA3 expression in the mammary gland [54]. Moreover, a previous study revealed that the expression of *TET2* and microRNA-200 is correlated, and their dysregulation can indirectly lead cancer stem cells to a luminal-cell-like state through the inhibition of protein kinase C zeta (PRKCZ) [55]. In fact, TET2 can also co-bind with ESRα at enhancer elements [58], making it crucial to efficient ER binding while acting as a facilitator of ER-chromatin interactions [59]. Additionally, in ER (+) breast cancer cells, TET2 predominantly binds to ER enhancer element sites, indicating that TET2 recruitment may depend on cell-specific factors [59,60]. Therefore, effective ER transcriptional activity may require TET2-dependent accumulation of 5-hmC at ER enhancer elements and subsequent control of cell cycle progression [59]. 

While TET2 and TET3 have the potential to influence estrogen receptors and luminal-like transformation in breast cancer, TET1 appears to be more associated with hormone-independent breast cancer. A study based on The Cancer Genome Atlas (TCGA) datasets revealed that almost 40% of patients with triple-negative breast cancer displayed overexpression of *TET1* [61]. Furthermore, metastatic breast cancer tissues and cell lines (including MDA-MB-231) exhibit lower *TET1* expression compared to non-invasive breast cancer samples and cell lines (including SkBr3) [62,63]. In line with these findings, we observed that the expression level of *TET1* is higher in ER (−) non-metastatic SkBr3 cells than in the MDA-MB-231 cell line, with the lowest expression levels seen in the MCF-7 cell line (Figure 2). In addition, *TET1* exists in at least two isoforms, a long form and a short form, with the latter lacking the CXXC DNA-binding domain [64]. The short isoform has been primarily detected in triple-negative breast cancer [65,66], and its expression corresponds to a worse prognosis [65,67]. The overexpression of the long isoform of *TET1* was found to be associated with the inhibition of the cell oncogenic phenotype in breast cancer [67]. On the contrary, decreased *TET1* expression in hormone-receptor-negative breast cancer is linked to its mislocalization within the cytoplasm [68]. In the hormone-dependent breast cancer cell line (BT474), TET1 can suppress invasion and adhesion [63], whereas in triple-negative breast cancer cells (MDA-MB-231), TET1 acts in an opposite manner, promoting cell proliferation and migration via activation of the oncogenic PI3K–mTOR pathway [61]. 

The alterations in the methylation profile in breast cancer may also occur in specific genes, including *ESR*, *PGR*, or *HER2*, affecting their expression, thus representing a different response to endocrine/hormonal therapies [69]. Moreover, because epigenetic modifications are prominently reversible [70,71] and TET enzymes are mainly responsible for mediating the active turnover of DNA methylation [6,7,8,70], changes in their expression, as well as in substrates and products of their activity, can serve as prognostic factors during cancer treatment. Furthermore, epigenetic modifications themselves can be of great importance during the therapy response [71,72,73], because epigenetic mechanisms are involved in several crucial cellular processes, such as apoptosis, cell replication, DNA repair, and the regulation of innate immunity or tumor suppression [57]. Moreover, it should be noted that alterations in the regulation of gene expression in breast cancer are particularly important mechanisms of resistance to hormonal therapy [74,75]. 

To date, few studies have explored the relationship between tamoxifen and epigenetics, particularly regarding the epigenetic resistance of breast cancer cells to tamoxifen therapy [76,77,78,79,80]. Therapeutic approaches using hormone antagonists target the dependence of breast cancer cells on estrogens to proliferate. The initial study by van Agthoven et al. indicated that estrogen-dependent breast cancer cells (ZR-75-1), treated with DNMT inhibitor (5-azacytydine, 5-aza) followed by 4-hydroxytamoxifen, developed tamoxifen resistance that was dependent on the 5-aza dose [78]. Interestingly, combined treatment with 5-aza and trichostatin A (an inhibitor of histone deacetylase) could epigenetically activate ER in triple-negative breast cancer cells (MDA-MB-231), thus making them sensitive to tamoxifen [81]. Hence, epigenetic alterations may play a significant role in tamoxifen’s mode of action. In vivo studies have demonstrated that rats receiving a diet supplemented with tamoxifen exhibited a decrease in global DNA methylation in the liver, manifesting as low cytosine methylation and decreased *DNMT1*, *DNMT3a*, and *DNMT3b* expression [82]. Changes in DNA methylation associated with DNMT1 were also observed in breast cancer cells treated with tamoxifen. Hypermethylation of Vestigial-like family member 4 (*VGLL4*)—a key gene taking part in the inhibition of breast cancer cells’ proliferation, migration, and tumor growth—served indirectly as an indicator of tamoxifen resistance [76]. In our study, we observed that 4-HT has a greater impact on *TET* mRNA expression in breast cancer cells than Endox. Moreover, breast cancer cells with expression of *ESR*, *PGR*, or *GPER* showed higher levels of *TET3* after 4-HT treatment, but in triple-negative cells (MDA-MB-231), *TET3* was deceased upon 4-HT supplementation (Figure 3). Changes in *TET3* expression were observed to correlate with variations in 5-hmC levels. Specifically, an increase in *TET3* expression was associated with elevated levels of 5-hmC in MCF-7 and SkBr3 cell lines (Figure 3G,I and Figure 6A,C), whereas in the MDA-MB-231 cell line, we found decreased *TET3* expression and a decreased 5-hmC level (Figure 3H and Figure 6B). Moreover, in the SkBr3 and MDA-MB-231 cell lines, similar changes were noticed in terms of *TET2* expression (Figure 3E,F and Figure 6B,C). These findings suggest that the changes in 5-hmC observed following 4-HT supplementation in breast cancer cells may be driven by TET3 or TET2 enzymes, and that these alterations are specific to *GPER* and *ESR* expression. Notably, 4-HT appears to exert a more specific impact on epigenetic changes, particularly on *TET3* expression, in cells expressing both receptors (MCF-7) (Figure 3D,G) compared to cells expressing only the GPER receptor (SkBr3) (Figure 3F,I). To our knowledge, there have been no studies to date investigating the impact of tamoxifen derivatives on active DNA demethylation. However, given that these derivatives can bind to estrogen receptors, and breast cancer behavior is modulated by hormonal influences, insights can be gleaned from research analyzing *TET* expression following exposure to estrogens. Studies conducted in vitro revealed that estrogen, either alone on combined with progesterone, can increase *TET3* expression in human endometrial epithelial cells (HES) and elevate TET3 protein levels in endometrial adenocarcinoma cell lines (AN3) [46]. Likewise, administration of estrogen results in the upregulation of *TET2* expression and 5-hmC levels in the MCF-7 cell line, whereas both were observed to decease following treatment with a selective estrogen receptor degrader (SERD) [83]. Moreover, the loss of *TET2* expression leads to tamoxifen resistance in vivo [54]. In turn, TET1 isoforms exhibit differential regulation in response to estrogen, but also to GnRH. The short isoform of TET1 is upregulated, whereas the expression of the long isoform is downregulated following exposure to either hormone [67]. Moreover, TET1 was also found as the upstream regulator of *GPER* expression in endometrial cancer cells [84]. However, in our study, tamoxifen derivatives appeared to have the least impact on *TET1* in breast cancer cells (Figure 3A–C). Among tamoxifen derivatives used in this study, 4-HT exhibited a more significant impact on 5-mC levels in all analyzed cell lines compared to Endox (Figure 5). Specifically, 4-HT induced an increase in 5-mC levels in cell lines expressing ESR and/or GPER receptors (MCF-7 and SkBr3, Figure 5A,C) and a decrease of this cytosine derivative level in the triple-negative cell line (MDA-MB-231, Figure 5B). The concurrent alterations observed in 5-mC and 5-hmC levels in ESR α and/or GPER-positive breast cancer cell lines following supplementation with anti-estrogens suggest that tamoxifen derivatives may influence both DNA methylation and active DNA demethylation processes. Based on previous studies, liganded estrogen receptor α (ESRα) can induce DNA methylation [85,86]. Furthermore, Li et al. reported that exposure of the MCF-7 cell line to estradiol resulted in an increase in DNMT3b protein levels, while DNMT1 and DNMT3a levels remained unchanged [87]. This observation suggests that the activated ESR α may contribute to de novo DNA methylation processes, potentially influencing transcriptional regulation and gene expression. Furthermore, treatment with the C29 compound, an inhibitor of ESR α, was also shown to increase DNMT3a levels in the SkBr3 cell line, providing additional evidence for the potential influence of antiestrogens on de novo DNA methylation [88].

## 4. Conclusions

Our study underscores the intricate role of estrogen receptors in modulating DNA methylation dynamics after tamoxifen treatment, highlighting their potential implications in gene regulation and cellular processes. Specifically, the impact of tamoxifen derivatives on DNA methylation dynamics, particularly through modulation of *TET3* expression, seems to rely on the presence of estrogen receptors. Moreover, because epigenetic alterations are highly reversible, they have become promising targets for anti-cancer therapy and improving treatment outcomes. The presented findings provide valuable insights from cell lines. To fully capture the complexities of tumors, where the microenvironment influences gene expression, future studies using animal models are recommended. This approach will enhance our understanding of the relationship between breast tumor subtypes and TET expression and function. 

## 5. Materials and Methods

### 5.1. Cell Culture

Four breast cancer cell lines with different ESR and GPER receptor expression (Table 1), MCF-7, MDA-MB-231, and SkBr3, were purchased from the Leibniz Institute DSMZ-German Collection of Microorganisms and Cell Cultures GmbH (Braunschweig, Germany). The MDA-MB-231 cell line, which lacks ESR α and GPER receptors, was selected as the control in the present study. Cells were cultured in standard RPMI 1640 medium (Capricorn Scientific GmbH, Ebsdorfergrund, Germany) supplemented with 5% feal bovine serum (FBS; HyClone, Cytiva, Logan, UT, USA), 1% (*v*/*v*) penicillin–streptomycin solution (Merck KGaA, Darmstadt, Germany), and 10 µg/mL insulin (Merck KGaA, Darmstadt, Germany). At 24 h before treatment with tamoxifen analogues, the medium was changed into RPMI 1640 without phenol red (Capricorn Scientific GmbH, Ebsdorfergrund, Germany) containing 5% (*v*/*v*) charcoal-stripped FBS (Capricorn Scientific GmbH, Ebsdorfergrund, Germany), 1% (*v*/*v*) penicillin–streptomycin solution (Merck KGaA, Darmstadt, Germany), and 10 µg/mL insulin (Merck KGaA, Darmstadt, Germany). Subsequently, the active derivatives of tamoxifen, 4-hydroxytamoxifen (Merck KGaA, Darmstadt, Germany) or endoxifen (Merck KGaA, Darmstadt, Germany), were added to the culture. The concentration of used reagents was estimated before the experiment based on a cell viability test (for MCF-7 and MDA-MB-231: 15 µM endoxifen and 15 µM 4-hydroxytamoxifen; for SkBr3: 10 µM endoxifen and 10 µM 4-hydroxytamoxifen), as described below. Every cell line was incubated with a specific reagent combination for 72 h. 

### 5.2. Cell Viability Assay

Cells were seeded on 96-well plates (0.15 × 10^5^ cells/well) and cultured in RPMI 1640 without phenol red (Capricorn Scientific GmbH, Ebsdorfergrund, Germany), 5% (*v*/*v*) charcoal-stripped FBS (Capricorn Scientific GmbH, Ebsdorfergrund, Germany), 1% (*v*/*v*) penicillin–streptomycin solution (Merck KGaA, Darmstadt, Germany), and 10 µg/mL insulin (Merck KGaA, Darmstadt, Germany). After growing to subconfluence, different concentrations of endoxifen (5–25 µM) and 4-hydroxytamoxifen (5–25 µM) were added to the wells and incubated for 72 h. Then, an MTT viability assay (Merck KGaA, Darmstadt, Germany) was performed. MTT (5 mg/mL in 1 × PBS) was prepared in the culture medium (final dilution, 1:10), 100 μL assay reagent was added to each well, and cells were incubated for three hours in a humidified atmosphere of 5% CO_2_ at 37 °C. The formazan crystals were dissolved in 100 μL isopropanol/0.04 N HCl (Merck KGaA, Darmstadt, Germany), followed by absorbance measurement at λ = 595 nm using the BioTek Fluorescence Microplate Reader (Lonza BioScience, Basel, Switzerland). The results were normalized to the controls.

### 5.3. RNA Isolation and RT-qPCR

First, 0.5 × 10^6^ cells were collected from each well, and then RNA was isolated using an RNA isolation kit (EURx, Gdańsk, Poland) according to the manufacturer’s protocol. The RNA concentration was measured using NanoDrop 2000c/2000 (ThermoScientific, Waltham, MA, USA). Following this, 0.5 μg total isolated RNA from each sample (in volume of 20 μL) was used for cDNA synthesis through reverse transcription using the High–Capacity cDNA Reverse Transcription Kit (ThermoScientific, Waltham, MA, USA), according to the procedures of the producer of the Mastercycler Nexus Gradient thermocycler (Eppendorf, Hamburg, Germany). To ensure the absence of genomic DNA contamination, negative controls were included in the reverse transcriptase reaction. The qRT–PCR complied with the Minimum Information for Publication of Quantitative Real–time PCR Experiments (MIQE) guidelines. Gene transcripts *TET1*, *TET2*, and *TET3* were analyzed through relative quantitative real-time RT–PCR (qRT–PCR) using PrimePCR Probe Assays (BioRad, Hercules, CA, USA) that include a pre-designed primer and probe sets specific to the target genes and SsoAdvanced Universal Probes Supermix (BioRad, USA). The expressions of target genes were normalized relative to two reference genes encoding β-actin (*ACTB*) and glucose–6–phosphate dehydrogenase (*G6PD*). The following assays were used: *TET1* (Unique Assay ID: qHsaCIP0026591), *TET2* (Unique Assay ID: qHsaCIP0029514), *TET3* (Unique Assay ID: qHsaCIP0027518), *ACTB* (Unique Assay ID: qHsaCEP0036280), and *G6PD* (Unique Assay ID: qHsaCEP0025798). The real-time PCR mixes in a volume of 10 μL were prepared from cDNA according to the standard procedures of SsoAdvanced Universal Probes Supermix (BioRad, Hercules, CA, USA) provided with the reagents set. Quantitative real-time PCR was carried out using the CFX Opus 96 Real-Time PCR System instrument (BioRad, Hercules, USA) with the following cycling parameters: 10 s at 95 °C followed by 45 repeats of 10 s at 95 °C, 30 s at 58 °C, and finally 1 s at 72 °C with acquisition mode. The standardizations of the reaction for each gene were performed to estimate the efficiency of amplification via standard curves.

### 5.4. DNA Isolation and HPLC with MS/MS

DNA isolation from cells has been described previously [89,90]. Briefly, cells were resuspended in ice-cold buffer B (10 mM Tris–HCl (Merck KGaA, Darmstadt, Germany), 5 mM Na_2_EDTA (Merck KGaA, Darmstadt, Germany) and 0.15 mM deferoxamine mesylate (pH 8.0; Merck KGaA, Darmstadt, Germany) in a 1:1 ratio. Sodium lauryl sulfate (SDS; Merck KGaA, Darmstadt, Germany) was then added (final concentration of 0.5% (*w*/*v*)), and samples were incubated at 37 °C for 30 min, followed by proteinase K (final solution concentration of 4 mg/mL; Merck KGaA, Darmstadt, Germany) addition and incubation at 37 °C for another 1.5 h. Following this, samples were extracted using phenol: chloroform: isoamyl alcohol (25:24:1) (Merck KGaA, Darmstadt, Germany) in a 1:1 ratio, and the aqueous phase was treated with a chloroform: isoamyl alcohol mixture (24:1). The supernatant was treated with cold 96% (*v*/*v*) ethanol to precipitate high-molecular-weight nucleic acids. The obtained precipitate was dissolved in Milli-Q-grade deionized water. The samples were mixed with 200 mM ammonium acetate containing 0.2 mM ZnCl_2_, pH 4.6 (1:1). Nuclease P1 (100 U; New England Biolabs, Hitchin, UK), tetrahydrouridine (Merck KGaA, Darmstadt, Germany), and 10 μg/sample were added to the mixture and incubated at 37 °C for 3 h. Next, 10% (*v*/*v*) NH_4_OH and 6 U shrimp alkaline phosphatase (rSAP; New England Biolabs, Hitchin, UK) were added to each sample and incubated for 1.5 h at 37 °C. Finally, all of the hydrolysates were ultrafiltered prior to injection to eliminate macromolecular compounds using AcroPrep Advance 96-Well Filter Plates 10 K MWCO (Pall Corporation, Port Washington, NY, USA) and centrifugation at 2000× *g* for 60 min at 4 °C.

The quantification of 5-methyl-2′-deoxycytidine (5-mdC) and 5-(hydroxymethyl)-2′-deoxycytidine (5-hmdC) by 2D-UPLC-MS/MS (Waters Corporation, Milford, CT, USA) was performed using the method reported in the previous papers [90,91]. Briefly, the molar concentration of modified deoxynucleoside was divided by the sum of molar concentrations of unmodified deoxynucleosides (dN), which served as a “secondary internal standard”, and it was expressed as the number of modified molecules per thousand (5-mC and 5-hmC), million (5-fC), or billion unmodified deoxynucleosides (5-caC), depending on their abundance.

### 5.5. Total Protein Extraction and Western Blot Analysis

Seventy-two hours after SERM treatment, the cells were washed three times with chilled phosphate-buffered saline (PBS; pH 7.3) and lysed with RIPA lysis buffer (EURx, Poland) supplemented with Pierce Phosphate Inhibitor Cocktail (ThermoFisher Scientific USA), Complete Protease Inhibitor Cocktail (Roche, Basel, Switzerland), and Viscolase (A&A Biotechnology, Gdańsk, Poland) on ice for 30 min. The total protein concentration was determined using the Pierce BCA Protein Assay Kit (ThermoFisher Scientific, USA). Samples were further processed as already described, with some minor modifications [92]. Twenty micrograms of the total protein lysate were resolved in 9% SDS-PAGE under reducing conditions and subsequently transferred onto a PVDF membrane (Merck Millipore, Carrigtwohill, Ireland). After one hour blocking in 5% skimmed milk in Tris-buffered saline (TBS) supplemented with 0.1% Tween 20 (TBS-T; Sigma-Aldrich, Steinheim, Germany) at room temperature, the membrane was probed overnight at 4 °C with a primary antibody (Table S1) diluted in 5% skimmed milk-TBS-T. After extensive washing with TBS-T, the membrane was incubated for 1 h at room temperature with a secondary antibody diluted in 1% skimmed milk-TBS-T. Following washing, signals from reactive bands were developed using the SuperSignal West Dura Extended Duration Substrate (ThermoFisher Scientific, USA) or SuperSignal West Pico PLUS Chemiluminescent Substrate (ThermoFisher Scientific, USA). See Supplementary Materials for the results. 

### 5.6. Statistical Analysis

The statistical analysis were conducted using Graph Pad Prism 9 (GraphPad Software, San Diego, CA, USA). All data are shown either as the fold change of the mean or as the mean +/− SEM. First, Pearson and d’Agostino omnibus normality tests were performed, followed by analysis using Student’s *t*-test (for Gaussian distribution) or the Mann–Whitney U-test (non-Gaussian distribution). A *p* < 0.05 was considered significant. 

## Figures and Tables

**Table 1:** Expression of receptors in analyzed breast cancer cell lines. ESR α—estrogen receptor α, ESR β—estrogen receptor β, PGR—progesterone receptor, GPER—G-protein-coupled estrogen receptor, HER2—human epidermal growth factor receptor 2.

Cell Line	ESR α	ESR β	PGR	GPER	HER2
MCF-7	+	+	+	+	−
MDA-MB-231	−	+	−	−	−
SkBr3	−	−	−	+	+

## Data Availability

The datasets used and/or analyzed during the current study are available from the corresponding authors upon reasonable request.

## Supplementary Materials

The following supporting information can be downloaded at: https://www.mdpi.com/article/10.3390/ijms25168561/s1.
