# Supplementary material for: Selective Estrogen Receptor Modulators’ (SERMs) Influence on TET3 Expression in Breast Cancer Cell Lines with Distinct Biological Subtypes
Source: Int J Mol Sci. 2024 Aug 6;25(16):8561. doi: 10.3390/ijms25168561 (PMC11354732; doi:10.3390/ijms25168561)
Supplement: Supplementary file 1 [file ijms-25-08561-s001.zip › ijms-3061746-supplementary.pdf]

Supplementary information:

Materials and methods:

Total protein extraction and Western blot analysis

Antigen/<br>Catalog No.	Type/<br>Clone (Symbol)	Dilution	Source
β-actin/<br>#3700	Mouse monoclonal (IgG2b)/<br>8H10D10	1:2000	Cell Signaling<br>Technology Inc.<br>(Beverly, USA)
TET1/<br>#SAB2700730	Mouse monoclonal (IgG2b)/<br>GT1462	1:1000	Sigma-Aldrich
TET2/<br>#MA5-31496	Mouse monoclonal (IgG2a)/<br>GT442	1:1000	ThermoFisher<br>Scientific Inc.
TET3/<br>#PA5-31860	Rabbit polyclonal	1:1000	ThermoFisher<br>Scientific Inc.

Table S1. Primary antibodies used in Western blotting.

Results:

Our goal was to perform Western Blot analysis to determine whether changes in gene expression of *TETs* are followed by the same changes in TET proteins level. However, we encountered significant challenges in performing the Western blot analysis for detecting the TET proteins. Despite multiple attempts and optimizations, we were unable to obtain results that were adequate for a reliable comparison with our gene expression analysis. Recognizing the importance of transparency and the need to document all efforts undertaken during our study, we have included the results of these analyses in the supplementary material.

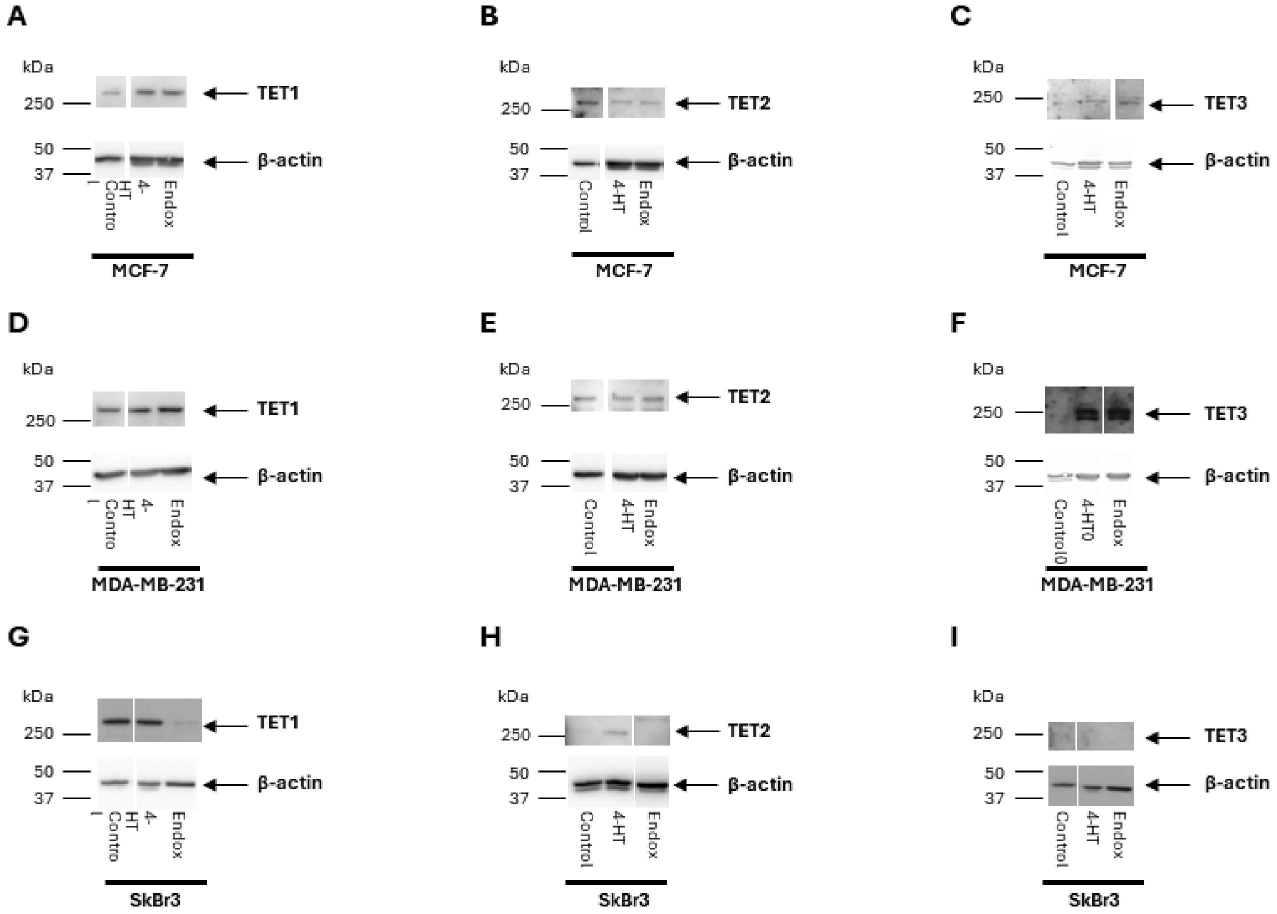

Figure S1. TET1 (Fig. S1A), TET2 (Fig. S1B), and TET3 (Fig. S1C) protein level in MCF-7 cell line; TET1 (Fig, S1D), TET2 (Fig. S1E) and TET3 (Fig. S1F) protein level in MDA-MB-231 cell line; TET1 (Fig. S1G), TET2 (Fig. S1H), and TET3 (Fig. S1I) protein level in SkBr3 cell line.
